# Cardiac Magnetic Resonance Fingerprinting: Technical Developments and Initial Clinical Validation

**DOI:** 10.1007/s11886-019-1181-1

**Published:** 2019-07-27

**Authors:** G. Cruz, O. Jaubert, R. M. Botnar, C. Prieto

**Affiliations:** 1 School of Biomedical Engineering and Imaging Sciences, King’s College London, 3rd Floor, Lambeth Wing, St Thomas’ Hospital, London, SE1 7EH UK; 2 Pontificia Universidad Católica de Chile Escuela de Ingeniería, Santiago, Chile

**Keywords:** Quantitative MRI, T1 mapping, T2 mapping, Cardiac fingerprinting, Myocardial tissue characterization

## Abstract

**Purpose of Review:**

Magnetic resonance imaging (MRI) has enabled non-invasive myocardial tissue characterization in a wide range of cardiovascular diseases by quantifying several tissue specific parameters such as T_1_, T_2_, and T2* relaxation times. Simultaneous assessment of these parameters has recently gained interest to potentially improve diagnostic accuracy and enable further understanding of the underlying disease. However, these quantitative maps are usually acquired sequentially and are not necessarily co-registered, making multi-parametric analysis challenging. Magnetic resonance fingerprinting (MRF) has been recently introduced to unify and streamline parametric mapping into a single simultaneous, multi-parametric, fully co-registered, and efficient scan. Feasibility of cardiac MRF has been demonstrated and initial clinical validation studies are ongoing. Provide an overview of the cardiac MRF framework, recent technical developments and initial undergoing clinical validation.

**Recent Findings:**

Cardiac MRF has enabled the acquisition of co-registered T_1_ and T_2_ maps in a single, efficient scan. Initial results demonstrate feasibility of cardiac MRF in healthy subjects and small patient cohorts. Current in vivo results show a small bias and comparable precision in T_1_ and T_2_ with respect to conventional clinical parametric mapping approaches. This bias may be explained by several confounding factors such as magnetization transfer and field inhomogeneities, which are currently not included in the cardiac MRF model. Initial clinical validation for cardiac MRF has demonstrated good reproducibility in healthy subjects and heart transplant patients, reduced artifacts in inflammatory cardiomyopathy patients and good differentiation between hypertrophic cardiomyopathy and healthy controls.

**Summary:**

Cardiac MRF has emerged as a novel technique for simultaneous, multi-parametric, and co-registered mapping of different tissue parameters. Initial efforts have focused on enabling T_1_, T_2_, and fat quantification; however this approach has the potential of enabling quantification of several other parameters (such as T_2_^*^, diffusion, perfusion, and flow) from a single scan. Initial results in healthy subjects and patients are promising, thus further clinical validation is now warranted.

## Introduction

In recent years, cardiovascular magnetic resonance (CMR) imaging has emerged as a key modality for non-invasive myocardial tissue characterization. In addition to the established late gadolinium enhancement MRI technique, this can be achieved by quantifying several tissue-specific parameters such as T_1_, T_2_, and T_2_^*^ relaxation times, and extracellular volume (ECV), enabling detection of focal and diffuse myocardial disease. T1 has been shown to be a sensitive parameter for detection of focal and diffuse fibrosis, whereas T_2_ and T_2_^*^ have been used to quantify edema/inflammation and iron deposition, respectively. CMR parametric mapping is recommended primarily for the assessment of amyloidosis, Anderson-Fabry disease, myocarditis, and myocardial infarction [1]. Moreover, research is currently ongoing to fully validate the performance of these imaging biomarkers in additional diseases such as cardiomyopathies, sarcoidosis, or transplant rejection [2, 3]. Simultaneous assessment of these parameters has recently gained interest to potentially improve diagnostic accuracy and enable further understanding of the underlying disease, for example using both T_1_ and T_2_ to differentiate acute/chronic myocardial infarction [4] or to better characterize inflammatory cardiomyopathy [5].

Current clinical methods for cardiac parameter mapping include modified look-locker inversion recovery (MOLLI) [6] for T_1_ and ECV, T_2_-prepared balanced steady state-free precession (T_2_prep bSSFP) for T_2_ [7] and multi-echo gradient echo (MEGE) [8] for T_2_^*^. While widely accepted in clinical practice, the accuracy of these methods can vary with sequence parameters and vendors; consequently, acquisition of local reference values is generally recommended. Acquisition of several parameter maps (in multiple slices, pre- and post-contrast agent injection) is generally recommended for myocardial tissue quantification [1]. However, this strategy requires multiple breath-holds, and thus the resulting maps are often not co-registered, hindering analysis and potentially leading to quantification errors. Moreover, among other confounding factors, T_2_ relaxation can affect quantification of T_1_, and vice versa, if both parameters are not simultaneously included in the signal model.

A new paradigm for unified quantitative parameter mapping, magnetic resonance fingerprinting (MRF), has been recently proposed. In contrast to conventional techniques, MRF provides simultaneous, co-registered, and multi-parametric mapping [9] from a single scan, allowing quantification of as many parameters as those included in the signal model. The latter promises not only quantification of several additional parameters of interest (e.g., diffusion, perfusion, CEST) [7], but also the potential correction of confounding factors (e.g., B_0_, B_1_, magnetization transfer) [9, 10••, 11], that may affect the accuracy, precision and reproducibility (multi-vendor, multi-site) of quantitative cardiac MRI. In MRF, sequence parameters (such as flip angle (FA) and repetition time (TR)) are continuously varied to sensitize the acquisition to tissue parameters of interest (such as T_1_ and T_2_). Cardiac MRF (cMRF) [10••] has been shown to provide simultaneous, co-registered T_1_ and T_2_ mapping in a single breath-hold. This review will cover the mechanisms of cardiac MRF, recent technical developments, and initial undergoing clinical validation.

### Conventional Cardiac Parametric Mapping

Cardiac T_1_ mapping (pre-contrast native T_1_ or post-contrast T_1_ for ECV) is usually performed with the modified look-locker inversion recovery (MOLLI) [6] type or saturation recovery single-shot acquisition (SASHA) [11] type acquisitions, which employ inversion recovery (IR) or saturation recovery (SR) preparation pulses, respectively. MOLLI uses IR pulses to achieve T_1_ contrast, sampling 8 images with different inversion time delays (TI), acquiring data over 11 heartbeats for a 5(3)3 acquisition; however it is sensitive to heart rate variations, magnetization transfer (MT), and T_2_ bias [12], among other confounding factors. SASHA relies on SR pulses for T_1_ encoding, acquiring one image without SR pulse and 9 images following SR pulses with different saturation delays (T_1_ contrasts) in 10 heartbeats. The SR pulse minimizes heart rate dependency and MT effects; however, the reduced dynamic range of the SR vs IR preparation leads to reduced precision in T_1_ for SASHA. MOLLI is characterized by high precision and a negative bias, whereas SASHA is characterized by high accuracy but lower precision [13].

Cardiac T_2_ mapping is commonly performed with T_2_-prepared balanced steady state-free precession (T_2_prep bSSFP) [7] or T_2_ gradient and spin echo (T_2_ GraSE) [14] sequences. T_2_prep bSSFP achieves T_2_ contrast by employing T_2_-prepared modules with different durations, sampling 3 T_2_-weighted images over 7 heartbeats; however, it is sensitive to the k-space sampling order (linear vs. centric), blood partial volume, and T_1_ relaxation time [15]. Since the majority of T_2_ encoding arises from the preparation pulse, other readouts are also suitable for T_2_-prepared mapping such fast low angle shot (FLASH) [16]. T_2_ GraSE mapping uses echo planar imaging readouts with varying effective echo times to the k-space center, sampling 9 T_2_-weighted images in 13 heartbeats; however, it is particularly sensitive to motion, T_2_^*^ decay, and signal to noise ratio (SNR) [17].

T_2_^*^ mapping is usually performed with multi-echo gradient echo (MEGE) sequences, acquiring 8 T_2_^*^-weighted images in a breath-hold; however, this approach is sensitive to magnetic susceptibility, blood partial volume, and SNR [18].

The methods outlined above, while widely accepted in clinical practice, are limited by a variety of confounding factors including inter-parameter dependency of T_1_/T_2_^(*)^. Moreover, current approaches require multiple acquisitions under several breath-holds to quantify multiple parameters. Notably, all of these methods use steady state sequences that sample discrete points along the T_1_/T_2_^(*)^ relaxation process, rely mostly on simplified exponential fit models, and map only one parameter at a time.

### Cardiac Magnetic Resonance Fingerprinting

Instead of sampling a few selected points along the relaxation curves, MRF attempts to capture the continuous transient state of the magnetization history. This is achieved with an acquisition and reconstruction framework that includes three main components: (1) variable pulse sequence sensitized to parameters of interest (e.g., T_1_ and T_2_); (2) highly undersampled acquisition (enabling high temporal resolution) that introduces incoherent image artifacts; (3) dictionary-based matching for multi-parametric map estimation. Pulse sequences with varying parameters (e.g., FA and TR) are used to generate unique signal evolutions (known as *fingerprints*) for each tissue, which is described by a given combination of parameters of interest. Highly undersampled k-space trajectories (typically spiral or radial) are employed to sample the transient signal evolution with a high temporal resolution (producing so called time-point images). In contrast to conventional parametric mapping, where a small number (~10) of fully-sampled (or moderately undersampled) time-point images are acquired, MRF acquires a large number (~1000) of highly undersampled time-point images. Instead of approximated exponential models, MRF uses the Bloch equations (or extended phase graphs [19, 20]) to simulate all possible tissue-specific signal evolutions for a particular sequence and range of parameters of interest (e.g., T_1_/T_2_ combinations), resulting in a so-called *dictionary* of fingerprints. Finally, the signal evolution for each pixel in the time-point image series is matched to the closest entry in the dictionary to retrieve the corresponding tissue-specific parameters of interest.

MRF was initially proposed for simultaneous T_1_, T_2_, M_0_, and B_0_ quantification in 2D brain imaging demonstrating reduced scan times relative to conventional methods. This framework promises several advantages over conventional mapping approaches: unification of multiple (co-registered) parameters in a single scan, natural extension to other parameters of interest (e.g., diffusion or MT) and to model corrections (e.g., B_0_ or slice profile correction), flexibility in designing optimal MRF sequences for specific applications (e.g., simultaneous T_1_ and T_2_ or simultaneous T_2_^*^ and diffusion), and higher parametric encoding efficiency, leading to reduced scan times [9, 21–23]. The main drawbacks for MRF are related to the complex acquisition and reconstruction process, which requires advanced sequence design and dictionary simulations to retrieve the underlying parameter values. Regardless, several recent technical developments have extended MRF to map various tissue and system-specific parameters (in addition to T_1_, T_2_, and M_0_) such as B_0_ [9], B_1_ [24], T_2_^*^ [25], diffusion [26], fat signal fraction [27], flow [28], and CEST [29].

Cardiac MRF was initially proposed for simultaneous T_1_, T_2_, and M_0_ mapping at 3 T [10••]. Similar to conventional mapping cardiac MRF relies on breath-held acquisitions to minimize respiratory motion, and therefore scan times are limited to achievable breath-hold durations (20–30 s). Electrocardiogram (ECG) triggering is used to minimize cardiac motion, limiting data acquisition to a small window (typically 100–250 ms) in the cardiac cycle, usually during mid-diastole. The need for ECG triggering requires several adaptations for cardiac MRF in comparison with conventional (static, e.g., brain imaging) MRF. In particular, variable magnetization preparation with interleaved IR and T_2_prep pulses are employed in cardiac MRF to increase sensitivity to T_1_ and T_2_ parameters. Moreover, whereas dictionaries can be computed once (per sequence) ahead of time for conventional MRF, cardiac MRF requires subject-specific dictionaries that incorporate information about the heart rate variability (RR interval) throughout the scan. Parametric maps can be derived from zero-filled reconstructions of highly undersampled time-point images in conventional MRF. However, in cardiac MRF the amount of acquired data is reduced (due to ECG triggering and breath-hold) and residual aliasing can propagate into errors in the parametric maps. Iterative reconstructions incorporating coil sensitivities (i.e., parallel imaging [30, 31]) and additional constraints (such as sparsity [32] or subspace modeling [33]) have been developed for highly accelerated MRF applications (such as cardiac MRF).

**Fig. 1:**
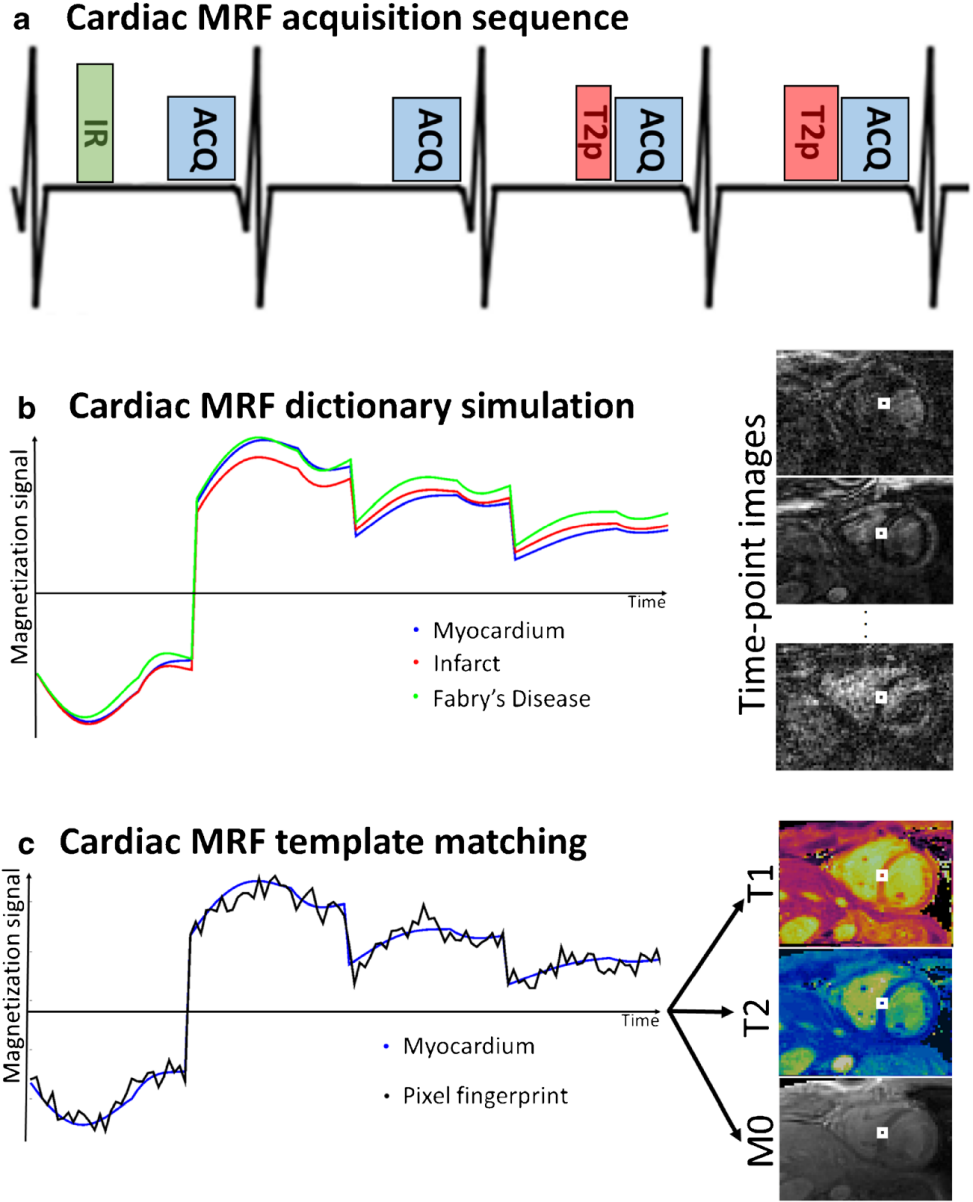
**a** Cardiac MRF acquires data with a triggered sequence. In each heartbeat an inversion recovery (IR), T_2_ preparation (T2p), or no preparation pulse is used before image acquisition (ACQ). This acquisition block is repeated several times during a breath-hold. **b** Patient-specific information (RR intervals) and sequence information (IR, T2p, FA, TR, etc) are used to simulate a dictionary of all the expected signal evolutions for all tissues of interest (here, three representative tissues are shown). The acquired data is reconstructed, producing a large series of time-point images corrupted with undersampling artifacts. **c** For each pixel in the time-point series, its temporal signal evolution (i.e., fingerprint) is extracted and compared with all the fingerprints in the dictionary via template matching. The highest correlation reveals the underlying tissue and corresponding tissue parameters (i.e., T_1_, T_2_, and M_0_)

The original cardiac MRF sequence employed a gradient echo readout, acquiring data over 16 heartbeats, divided into 4 blocks of 4 heartbeats each [10••]. In each block, data is acquired with the following magnetization preparation scheme: IR pulse, no preparation, T_2_prep (40 ms), and T_2_prep (80 ms) (Fig. 1a). Different blocks have different inversion time delays (TI) following the IR pulse 21 ms, 100 ms, 250 ms, and 400 ms. In each heartbeat, a set of 48 time-point images is acquired with a highly undersampled, golden angle [34], variable density spiral trajectory, leading to an acquisition window of ~250 ms and a total scan time of ~16 s (corresponding to 768 total time-point images). FA and TR are varied between approximately 5–15° and 5–6 ms, respectively. The use of relatively low flip angles help in reducing potential errors from B_1_ errors. The ECG log containing relative timings of preparation and imaging pulses is recorded during each scan and used to simulate a scan-specific dictionary (Fig. 1b) that accounts for patient-specific heart rate variabilities. T_1_ values in the range 50–5000 ms and T_2_ values in the range 6–500 ms are simulated to generate a dictionary of ~15000 entries. Time-point images are reconstructed in [35] with an iterative multi-scale algorithm to suppress residual aliasing artifacts. Finally, dictionary-based template matching is applied pixel by pixel to generate T_1_, T_2_, and M_0_ maps (Fig. 1c).

Cardiac MRF was initially compared with conventional MOLLI and T_2_prep bSSFP mapping techniques, demonstrating similar parametric map quality, albeit from a single scan. Cardiac MRF accuracy was similar to MOLLI, whereas a small negative bias was observed when compared with T_2_prep bSSFP; precision for cardiac MRF and conventional methods was similar. Cardiac MRF presented consistent values across varying heart rates (in simulations) and even in reduced number [4–9, 10••, 11, 12] of heartbeats (in vivo) when combined with advanced iterative reconstructions. Initial cardiac MRF results presented some variability in T_1_ and T_2_ maps due to errors coming from B_0_, B_1_, slice profile, cardiac motion, residual aliasing, and noise amplification. Additionally, coverage was limited to one slice and only T_1_ and T_2_ myocardial quantification was explored.

### Cardiac MRF: Technical Developments

One of the main obstacles for widespread clinical usage of parametric mapping have been confounding factors that affect the accuracy and reproducibility of these techniques. Cardiac MRF is also sensitive to hardware imperfections and undesirable effects from the underlying MR physics. However, cardiac MRF offers a higher flexibility to design sequences that could minimize these errors. Initial research [36•] into confounding factors for cardiac MRF (using gradient echo readouts) has revealed dependencies on slice profile, B_1_, and efficiency of preparation pulses (IR and T_2_prep). Slice profile errors introduce a negative bias in T_1_ and a positive bias in T_2_, B_1_ errors are closely connected with T_2_ variations and preparation pulse efficiency errors can lead to negative bias in T_1_ and positive bias in T_2_. Although these (and more) model corrections can be incorporated into the dictionary simulation, the computational time increases exponentially with the number of parameters. The same study investigated the sensitivity to the above confounding factors for a set of candidate sequences with varying combinations of preparation pulses and flip angle patterns. The best performing sequences used varying IR and T_2_prep pulses, with flip angles limited to 25°. Considerable errors were observed with high flip angle sequences without any corrections (5–10% and 4–15% for T_1_ and T_2_, respectively). Errors were reduced (1–2% and 3–5% for T_1_ and T_2_, respectively) when multiple corrections (for slice profile, B_1_ and preparation pulse efficiency) were incorporated in the model. Small flip angles (< 25°) sequences required less corrections. Optimal sequence design has been studied for MRF [22, 37], but not for ECG-triggered, preparation pulse-based cardiac MRF. Moreover, the investigation of confounding factors in MRF has revealed many effects that can bias results: intra-voxel dephasing [38], incidental spoiler diffusion [39], magnetization transfer [40], B_0_ [9], B_1_ [24], slice profile [41, 42], motion [43–45], inversion efficiency [46], partial volume and multi-compartment signal models [47, 48], and residual aliasing artifacts [49, 50].

Multi-slice coverage (basal, mid, and apical) is recommended in myocardial characterization protocols. This has motivated the development of simultaneous multi-slice (SMS) cardiac MRF [51]. SMS uses multiband RF pulses to simultaneously excite several slices with different RF phases; the signal from different slices can then be separated in the reconstruction and template matching steps [52]. SMS was combined with iterative low-rank reconstructions [33, 53] to further improve the accuracy and precision of the parameter maps. This approach enabled three slices to be simultaneously acquired in a 16-heartbeat breath-hold. When compared with MOLLI in healthy subjects, SMS cardiac MRF resulted in T_1_ values approximately 90 ms higher (1320 ± 52 ms vs. 1242 ± 33 ms), likely due to additional model corrections present in cardiac MRF that are not considered for MOLLI, namely slice profile and relaxation during preparation pulses (leading to imperfect inversions). However, SMS cardiac MRF T_1_ values were still underestimated relative to literature SASHA values, likely due to remaining un-modeled confounding factors such as magnetization transfer [40] or B_1_ errors [24]. When compared with T_2_prep FLASH, SMS cardiac MRF resulted in a negative bias of approximately 6 ms (31.7 ± 1.4 ms vs. 37.7 ± 2.3 ms); again this discrepancy is possibly due to remaining confounding factors such as spoiler gradient–induced diffusion [39], magnetization transfer [40], or through-plane motion [43]. A desirable extension for complete myocardial characterization would be 3D whole-heart coverage. Breath-holding is not possible in this case and respiratory motion compensation is required. Preliminary work has shown the feasibility of 3D free-breathing cardiac MRF for whole-heart simultaneous T_1_ and T_2_ mapping [54].

Fat quantification is a useful biomarker to characterize infarction and fatty infiltration [55]; additionally, fatty infiltrations can introduce bias in myocardial tissue characterization [56]. Cardiac MRF has been extended to simultaneous T_1_, T_2_, and fat fraction quantification [57] at 1.5T. To achieve this, a 3-point Dixon [58, 59] acquisition is incorporated into the cardiac MRF framework to enable water/fat separation. Instead of spiral sampling, a radial trajectory is used to efficiently sample the required Dixon echo times and a local low-rank tensor regularized reconstruction [60] is used to cope with the higher acceleration factors. Dixon cardiac MRF was compared with MOLLI and SASHA in healthy subjects, demonstrating T_1_ values of 1033 ± 51 ms, 1020 ± 66 ms, and 1126 ± 121 ms, respectively; for T_2_, Dixon cardiac MRF and T_2_ GraSE values were 43.2 ± 4.4 ms and 51.2 ± 4.9 ms, respectively; finally a fat fraction of 8% was measured in healthy subjects with this method. An alternative cardiac MRF approach for simultaneous T_1_, T_2_, and water/fat separation has also been developed using a Rosette trajectory [61]. This trajectory crosses the k-space center several times during readout. Fat or water suppression can be achieved by demodulating the acquired k-space data by the water or fat precession frequencies, respectively. Preliminary results from Rosette cardiac MRF (at 3T) produced T_1_ and T_2_ values of 1329 ± 22 ms and 30.7 ± 2.1 ms, respectively, in addition to water and fat separated proton density maps.

Functional CINE MR imaging is a gold standard technique for the visualization of wall motion abnormalities and quantification of ejection fraction. Motion resolved (MORE) cardiac MRF has been proposed [62] in an effort to provide simultaneous T_1_, T_2_, and CINE information. In contrast to triggered cardiac MRF, in MORE cardiac MRF data is continuously acquired with a radial bSSFP readout and then retrospectively gated into different cardiac phases. IR pulses regularly interrupt the acquisition to provide T_1_ encoding and the variable flip angle bSSFP readout provides T_2_ encoding. This approach produces T_1_ and T_2_ parameter maps in sixteen cardiac phases which could be used for joint tissue characterization and functional assessment. A similar approach, named CINE-MRF [63], also acquires data continuously with a spiral gradient echo readout and retrospectively reconstructs multiple cardiac phases. CINE-MRF estimates the motion within the cardiac cycle via image registration and uses this information to further improve the accuracy and precision of the cardiac resolved T_1_ and T_2_ maps. Twenty-five cardiac phases were obtained in this fashion and single-slice ejection fraction was found in agreement with standard CINE measurements. Another related promising solution for motion resolved parametric mapping is the recently proposed multitasking (MTT) technique [64]. MTT aims to capture all the dynamics of a cardiac MR protocol in a unified reconstruction. Examples of these dynamics include respiratory and cardiac motion, T_1_ and T_2_ contrast, and contrast perfusion. MTT has been applied for free-breathing, ECG-free T_1_/T_2_ mapping, acquiring data with a FLASH readout regularly interrupted by hybrid T_2_IR pulses. Respiratory and cardiac motion information is derived from the data itself with this approach and data is reconstructed within a low-rank tensor framework that naturally exploits correlations between different dynamic dimensions. This enables MTT to produce T_1_ and T_2_ maps in any respiratory or cardiac phase.

### Cardiac MRF: Initial Ongoing Clinical Validation

The accuracy, precision, and reproducibility of cardiac MRF has been validated in 50 healthy subjects at 1.5T [65]. Three slices (base, mid, apex) were acquired with cardiac MRF in this study. The mid slice was acquired twice during the same scan session to evaluate reproducibility. Results showed similar values (964 ± 71 ms and 978 ± 33 for cardiac MRF and MOLLI, respectively; 41.2 ± 4.2 ms and 46.6 ± 2.7 ms for cardiac MRF and T_2_prep bSSFP, respectively) and excellent reproducibility (via the intra-class coefficient) for all methods. Blinded evaluation of the parametric maps revealed a preference for cardiac MRF in terms of sharpness of myocardial anatomy, absence of artifacts, and overall image quality. Inter-site reproducibility of cardiac MRF has also been evaluated in a separate study [66]. Nine healthy subjects were scanned in two different sites using the same 3T scanner model [66]. MOLLI scans were performed at both sites, site A acquired T_2_prep bSSFP data whereas site B acquired T_2_prep FLASH data. Excellent reproducibility of cardiac MRF was observed between sites (1354 ± 40 ms and 1346 ± 38 ms (T_1_), 29.7 ± 2.8 ms and 29.6 ± 4.2 ms (T_2_), for sites A and B, respectively). Cardiac MRF values were generally higher than MOLLI (1220 ± 39 ms and 1208 ± 26 ms, for sites A and B) and considerably lower than T_2_prep bSSFP and T_2_prep FLASH (41.6 ± 1.6 ms and 38.2 ± 2.1 ms, for sites A and B, respectively).

Encouraging results in healthy subject cohorts warranted further evaluation of cardiac MRF in patients. The first reported cardiac MRF clinical application was for heart transplant patients where graft rejection causes edema that can be measured with T_2_. Thirteen patients and five healthy controls were scanned with 2D cardiac MRF in basal and mid slices; patient biopsies were performed to detect graft rejection [67]. No rejection was observed in patients. Healthy controls showed slightly reduced T_1_ and T_2_ values relative to patients with both cardiac MRF and conventional mapping techniques. Consistent values were obtained in transplant patients: 1252 ± 53 ms and 1260 ± 63 ms in T_1_ pre-contrast, 41.7 ± 4.8 ms and 40.9 ± 1.9 ms in T_2_ pre-contrast, and 629 ± 91 ms and 633 ± 63 ms in T_1_ post-contrast, for cardiac MRF and MOLLI/T_2_prep bSSFP, respectively. More recently, cardiac MRF has been evaluated for inflammatory cardiomyopathy [68] where both T_1_ (stronger in acute phase) and T_2_ (stronger in chronic phase) are relevant biomarkers [5]. Furthermore, this disease is common in patients with implantable cardioverter defibrillators that can cause considerable image artifacts. Twenty-four patients with suspected inflammatory cardiomyopathy (4 of them with implants) were scanned with 2D cardiac MRF at 1.5T; no additional model corrections (e.g., slice profile and inversion efficiency) were used. Cardiac MRF T_1_ values were similar to MOLLI (1028 ± 64 ms and 1019 ± 53 ms, respectively) whereas T_2_ values were slightly higher than T_2_prep bSSFP (52.8 ± 3.8 ms and 49.3 ± 3.1 ms, respectively). Images from patients with implants were scored on the presence of artifacts in the left ventricle (on the scale 0–4, 4 corresponding to no apparent artifacts), revealing a preference for cardiac MRF in both T_1_ (3.0 ± 0.8 vs. 2.3 ± 1.0) and T_2_ (2.8 ± 1.0 vs. 1.5 ± 1.0) parameter maps with respect to conventional MOLLI and T2prep bSSFP. Cardiac MRF has recently been applied in patients with hypertrophic cardiomyopathy (HCM) in two studies at 1.5T [69] and 3T [70]. The study at 1.5T compared 2D cardiac MRF with MOLLI and T_2_prep bSSFP, pre- and post-contrast, in 6 patients and 12 healthy subjects (without extra model corrections for cardiac MRF). Despite some differences between cardiac MRF and conventional methods, both approaches found significant differences between healthy subjects and HCM patients for pre-contrast T_1_ (cardiac MRF 921 ± 65 ms and 1017 ± 31 ms, respectively; MOLLI 996 ± 23 ms and 1057 ± 76 ms, respectively) and ECV (cardiac MRF 25 ± 3% and 37 ± 4%, respectively; MOLLI 21 ± 2% and 32 ± 2%, respectively). No significant differences between healthy subjects and HCM patients were observed in pre-contrast T_2_ (cardiac MRF 43.7 ± 4.3 ms and 45.0 ± 5.8 ms, respectively; T_2_prep bSSFP 43.7 ± 1.8 ms and 45.1 ± 3.7 ms, respectively). Interestingly, significant differences between healthy subjects and HCM patients (32.1 ± 2.6 ms and 35.9 ± 3.2 ms, respectively) were also observed for post-contrast T_2_ with cardiac MRF. The study at 3T compared cardiac MRF with MOLLI and T_2_prep FLASH, pre- and post-contrast, in 23 HCM patients. General image quality and artifact presence was evaluated in 1–5 Likert scale (5 corresponding to excellent quality). This analysis revealed a preference for conventional mapping methods pre-contrast and similar quality post-contrast. Cardiac MRF T_1_ values were higher than MOLLI pre- (1397 ± 40 ms vs. 1252 ± 25 ms) and post- (521 ± 46 ms vs. 488 ± 36 ms) contrast; significant differences were also observed for cardiac MRF and MOLLI derived ECV (27.6 ± 2.7% vs. 23.4 ± 2.0%, respectively). Cardiac MRF T_2_ values were also considerably lower than T_2_prep FLASH (29.0 ± 2.7 ms vs. 39.7 ± 1.7 ms, respectively). A summary of the parameter values determined in these studies is compiled in Table 1.

**Table 1:** T_1_, T_2_ (in ms) and ECV (in %) values from initial ongoing studies performed in healthy subjects and patients, comparing cardiac MRF and conventional methods

Study	cMRF T1 (pre-constrast)	Conventional T1 (pre-contrast)	cMRF T2 (pre-contrast)	Conventional T2 (pre-contrast)	cMRF T1 (post-constrast)	Conventional T1 (post-contrast)	cMRF ECV	Conventional ECV
Original cMRF [10••] 3T	1213 ± 75	(MOLLI) 1257 ± 61	34.9 ± 3.8	(T_2_prep bSSFP) 41.6 ± 5.0	N/A	N/A	N/A	N/A
cMRF model corrections [36•] 3T	1323 ± 47	(MOLLI) 1227 ± 30	37.2 ± 4.4	(T_2_prep FLASH) 38.4 ± 3.1	N/A	N/A	N/A	N/A
SMS cMRF [51] 3T	1320 ± 52	(MOLLI) 1242 ± 32	31.7 ± 1.4	(T_2_prep FLASH) 37.7 ± 2.3	N/A	N/A	N/A	N/A
Dixon cMRF [57] 1.5T	1033 ± 51	(MOLLI)/(SASHA) 1020 ± 66/1126 ± 121	43.2 ± 4.4	(T_2_ GraSE) 51.2 ± 4.9	N/A	N/A	N/A	N/A
Rosette cMRF [61] 3T	1329 ± 22	N/A	30.7 ± 2.1	N/A	N/A	N/A	N/A	N/A
MORE MRF [62] 1.5T	1138 ± 69	(MOLLI)/(SASHA) 1025 ± 37/1132 ± 100	44 ± 5	(T_2_ GraSE) 52 ± 5	N/A	N/A	N/A	N/A
CINE MRF [63] 3T	~1410	(MOLLI) ~1220	~31	(T_2_prep FLASH) ~39	N/A	N/A	N/A	N/A
50 healthy subject study [65] 1.5T	964 ± 72	(MOLLI) 979 ± 34	41.2 ± 4.2	(T_2_prep bSSFP) 46.6 ± 2.7	N/A	N/A	N/A	N/A
Inter-site study [66] Site A, 3T	1354 ± 40	(MOLLI) 1220 ± 39	29.7 ± 2.8	(T_2_prep bSSFP) 41.6 ± 1.6	N/A	N/A	N/A	N/A
Inter-site study [66] Site B, 3T	1346 ± 38	(MOLLI) 1208 ± 26	29.6 ± 4.2	(T_2_prep FLASH) 38.2 ± 2.1	N/A	N/A	N/A	N/A
Heart transplant patient study [67] 3T	1252 ± 53	(MOLLI) 1261 ± 63	41.7 ± 4.8	(T_2_prep bSSFP) 40.9 ± 1.9	620 ± 81	(MOLLI) 621 ± 48	N/A	N/A
Inflammatory cardiomyopathy patient study [68] 1.5T	1028 ± 64	(MOLLI) 1019 ± 53	52.8 ± 3.8	(T_2_prep bSSFP) 49.3 ± 3.1	N/A	N/A	N/A	N/A
Hypertrophic cardiomyopathy patient study [69] 1.5T	1017 ± 31	(MOLLI) 1057 ± 76	45 ± 6	(T_2_prep bSSFP) 45 ± 4	315 ± 43	(MOLLI) 336 ± 34	37 ± 4	32 ± 2
Hypertrophic cardiomyopathy patient study [70] 3T	1397 ± 40	(MOLLI) 1252 ± 25	29.0 ± 2.7	(T_2_prep FLASH) 39.7 ± 1.7	521 ± 46	(MOLLI) 488 ± 36	27.6 ± 2.7	23.4 ± 2.0

### Summary and Future Directions

Cardiac magnetic resonance fingerprinting has been recently introduced, enabling simultaneous and co-registered T_1_ and T_2_ mapping in a single breath-hold. Initial studies in healthy subjects and patients have shown that cardiac MRF achieves comparable parametric map quality to conventional methods (in reduced scan time); however, several biases have been detected. Reduction of bias due to sequences (e.g., MOLLI vs SASHA, T_2_prep bSSFP vs T_2_ GraSE), vendors and confounding factors has been an important research focus over the last decade to achieve truly quantitative cardiac MR imaging. MRF could contribute to this goal due to its flexibility to incorporate model corrections, which already has been shown to remove some of these confounding factors. In theory, all known factors from the subject’s physiology, MR physics and hardware could be incorporated into the cardiac MRF framework. In practice, this would lead to infeasible dictionary computation times; however, recent advances in machine learning for Bloch simulations could dramatically reduce computational requirements [71]. Such developments should also facilitate the extension of current cardiac MRF to additional parameters of interest such as T_2_^*^, magnetization transfer, quantitative susceptibility, diffusion, multi-compartment, and perfusion. Multi-parametric analysis (e.g., radiomics) of this new wealth of myocardial tissue information could potentially improve diagnostic accuracy and enable further understanding of the underlying disease. Clinical validation of cardiac MRF is still in its very early stage; however, promising results have been demonstrated in heart transplant patients, and patients with suspected inflammatory and hypertrophic cardiomyopathies. Further clinical validation is now warranted to evaluate the reproducibility of this technique and its potential to eventually replace currently available mapping techniques. Following in-depth clinical validation, cardiac MRF could be clinically available in the next three years. Currently, several technical developments are still needed to fully exploit the potential of cardiac MRF to provide truly quantitative myocardial characterization for a wide range of tissue parameters. Additional validation is required before widespread clinical adoption; however, cardiac MRF holds several promises that may significantly impact the field of quantitative cardiac MRI in the future.
